# Accusafe guidewire for atrial septal puncture: dual-imaging safety and efficacy: a case report

**DOI:** 10.3389/fcvm.2026.1652997

**Published:** 2026-02-09

**Authors:** Qian Nie, Haseeb Sattar, Ailing Huang, Hongcai Zhang, Jue Zhao, Delai Zhang, Xie Wen

**Affiliations:** 1 Department of Cardiology, Affiliated Hospital of Chengdu University of Traditional Chinese Medicine, Chengdu, Sichuan, China; 2 Department of Medicine, Urban Vocational College of Sichuan, Chengdu, China; 3 Department of Cardiology, Chengdu Pidu District Hospital of Traditional Chinese Medicine, Chengdu, China

**Keywords:** atrial fibrillation, atrial septal puncture, echocardiography, fluoroscopy, guidewire

## Abstract

A 74-year-old female with nonvalvular atrial fibrillation and heart failure, on rivaroxaban 20 mg/day, presented for left atrial appendage (LAA) closure due to recurrent hematomas despite anticoagulation therapy. This case report describes an innovative atrial septal puncture (ASP) technique utilizing the Accusafe guidewire (Synaptic Medical, Beijing, China) under combined fluoroscopic and intracardiac echocardiography (ICE) guidance. The procedure was successful, demonstrating that this method may enhance procedural safety and precision, particularly in complex cases such as LAA closure, atrial fibrillation ablation, and MitraClip procedures.

## Clinical case illustration

**Figure 1:**
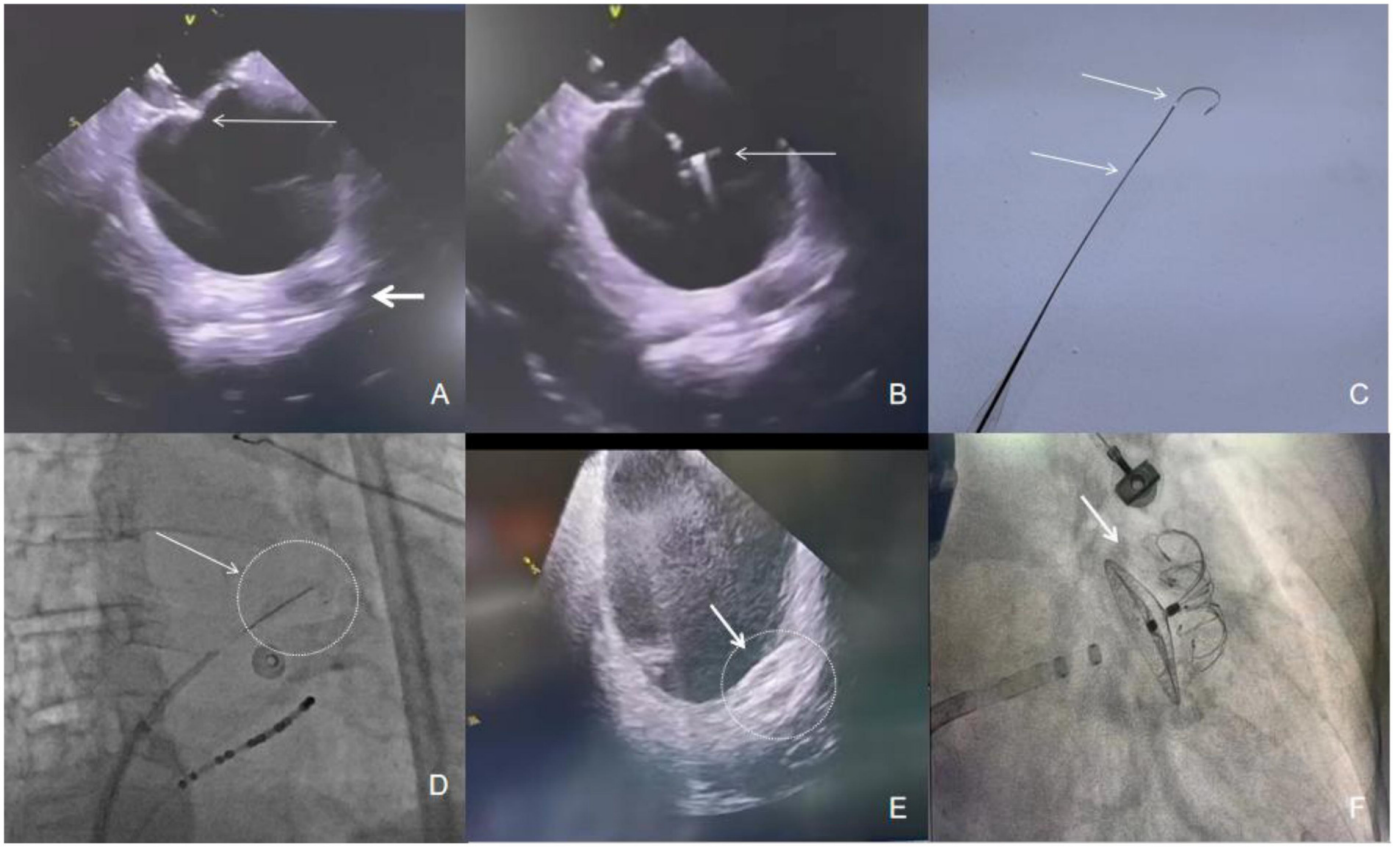
**(Panel A)** Small arrow: ICE shows “tenting” in atrial septal, big arrow: LAA, which indicates the posterior position of the atrial septal under ICE. **(Panel B)** Small arrow: the head of the Accusafe guidewire in the left atrium. **(Panel C)** Shape of guidewire and radiopaque period. **(Panel D)** Arrow and Circle: The tip and proximal period of the guidewire are radiopaque to indicate the position of the wire under x-ray. **(Panel E)** Arrow and Circle: ICE shows Lamax device was firmly planted in LAA. **(Panel F)** Arrow: Final shape of Lamax device in LAA by fluoroscopy.

A 74-year-old female with long-standing persistent nonvalvular atrial fibrillation (CHA_2_DS_2_-VASc 5, HAS-BLED 4), hypertension, and congestive heart failure (LVEF 45%) was scheduled for LAA closure. Recurrent subcutaneous hematomas from chronic rivaroxaban therapy necessitated a strategy to minimize procedural anticoagulation. Pre-procedural transesophageal echocardiography (TEE) revealed significant left atrial enlargement (49 × 54 mm), displacing the interatrial septum and obscuring standard fluoroscopic landmarks. This anatomy increased the difficulty and perforation risk of a traditional transseptal approach. Under synchronized fluoroscopy and 2D intracardiac echocardiography (ICE) guidance (Johnson & Johnson, SNDSTR10), the Accusafe guidewire was advanced through an 8Fr sheath (Johnson & Johnson) positioned at the optimal tenting site (Figures 1C,D). Real-time ICE allowed millimeter-scale adjustment to a posterior-inferior puncture trajectory (Figure 1A), achieving first-pass (single-attempt) septal penetration (Figure 1B). The guidewire's shape-memory U-tip prevented overshooting, mitigating risk of injury to adjacent structures. The ASP step itself was performed without the use of contrast agent. Post-puncture, ICE confirmed stable wire co-axiality with the LAA ostium, facilitating single-pass deployment of a LAMAX device (Figures 1E,F). The total procedure time was 38 min under local anesthesia, with only 2.1 min of fluoroscopy. Final ICE and angiography confirmed optimal device position without leak. Immediate post-procedural assessment showed no hemodynamically significant residual atrial septal defect. The patient resumed aspirin on postoperative day 1 and was discharged on day 3 without bleeding or thromboembolic events, demonstrating the technique's potential to mitigate bleeding risks while effectively preventing stroke. Longer-term follow-up data are being collected.

## Technical overview

Traditional ASP using a Brockenbrough (BRK) needle carries risks related to limited control and operator dependence. Our approach integrates the Accusafe guidewire—a nitinol device with a sharp, steerable tip and U-shaped memory retention.

### Device characteristics

The guidewire features a sharp tip for penetration, a 3 mm thickened proximal segment (0.035″) for anchorage, and a distal taper (0.018″) for flexible navigation. Compared to super-stiff guidewires, it offers slightly less support but greater safety due to its shape-memory tip and tapered design.

### Dual imaging guidance

Synchronized fluoroscopy and ICE enable real-time assessment of septal anatomy. Without ICE, transthoracic echocardiography can provide basic guidance, as the metallic guidewire produces distinct acoustic artefacts, though ICE offers superior resolution.

### Targeted puncture and position verification

The guidewire is advanced through the sheath to the septum. Correct left atrial position post-puncture can be confirmed via fluoroscopic observation, blood aspiration, pressure monitoring, or contrast injection.

### Safety mechanism

The self-reconfiguring J-tip post-puncture eliminates device exchanges, reducing risks of air embolism, tamponade, and wall trauma.

## Clinical advantages

### Precision and safety

ICE allows direct septal visualization, reducing contrast or TEE need. The shape-memory design helps prevent overshooting, potentially reducing pericardial tamponade risk compared to historical BRK needle data.

### Versatility

The technique preserves septal integrity for multiple sheath insertions and adapts to complex anatomies via real-time, imaging-guided puncture optimization.

### Efficiency

The single-step puncture-wire integration avoids needle-guidewire exchanges, potentially shortening procedure time.

## Discussion

### Patient selection and anatomic considerations

The Accusafe guidewire was selected for this patient's complex anatomy, including long-standing persistent atrial fibrillation, significant left atrial enlargement, and a septal aneurysm, which displaced the septum and obscured fluoroscopic landmarks (1, 2). This technique appears well-suited for such scenarios, where real-time imaging facilitates accurate puncture. A relative contraindication is a history of surgical septal repair, where resilient patch material may increase puncture difficulty (3).

### Safety and efficacy in context

This single case successfully achieved first-pass puncture and LAA closure without complications. The 38 min procedure suggests potential efficiency, though definitive time savings cannot be claimed. The shape-memory mechanism may reduce overshooting risk compared to historical BRK needle tamponade rates (4, 5). While not paradigm-shifting, this tool is a promising innovation. Future multicenter studies are needed to validate its broader applicability and safety, particularly in complex, high-risk anatomy as demonstrated in this case. Furthermore, this report details the first published use of the Accusafe guidewire in conjunction with the domestic LAMAX occluder system, offering practical insights for this specific device combination.

## Conclusion

This case demonstrates that the Accusafe guidewire under dual ICE/fluoroscopy guidance can facilitate safe and precise ASP in general anatomy, achieving successful first-pass puncture and LAA closure with a short procedure time and no complications. Its integrated design may enhance safety by mitigating overshoot risk and eliminating device exchange. This initial experience in a patient with complex anatomy confirms the technique's feasibility and suggests potential advantages for a broad range of cardiologists performing left atrial interventions, including in electrophysiology and structural heart procedures. Further studies are warranted to establish its broader applicability and safety profile.

## Data Availability

The original contributions presented in the study are included in the article/Supplementary Material, further inquiries can be directed to the corresponding author.
